# Willingness to Receive COVID-19 Vaccination Among People Living With HIV and AIDS in China: Nationwide Cross-sectional Online Survey

**DOI:** 10.2196/31125

**Published:** 2021-10-21

**Authors:** Xiaojie Huang, Maohe Yu, Gengfeng Fu, Guanghua Lan, Linghua Li, Jianzhou Yang, Ying Qiao, Jin Zhao, Han-Zhu Qian, Xiangjun Zhang, Xinchao Liu, Xia Jin, Guohong Chen, Hui Jiang, Weiming Tang, Zixin Wang, Junjie Xu

**Affiliations:** 1 Infectious Disease Department Beijing Youan Hospital Capital Medical University Beijing China; 2 Tianjin Center for Disease Control and Prevention Tianjin China; 3 Jiangsu Provincial Center for Disease Control and Prevention Nanjing China; 4 Guangxi Center for Disease Control and Prevention Nanning China; 5 Infectious Disease Department Guangzhou Eighth People's Hospital Guangzhou Medical University Guangzhou China; 6 Department of Preventive Medicine Changzhi Medical College Changzhi China; 7 Infectious Disease Department The Second Hospital of Huhhot Huhhot China; 8 Shenzhen Center for Disease Control and Prevention Shenzhen China; 9 SJTU-Yale Joint Center for Biostatistics and Data Sciences Shanghai Jiao Tong University Shanghai China; 10 Department of Public Health University of Tennessee Knoxville, TN United States; 11 Infectious Disease Department Peking Union Medical College Hospital Beijing China; 12 AIDS Healthcare Foundation China Beijing China; 13 Jockey Club School of Public Health and Primary Care Faculty of Medicine The Chinese University of Hong Kong Hong Kong China; 14 STD Prevention and Control Department University of North Carolina Project-China Guangzhou China; 15 Clinical Research Academy Peking University Shenzhen Hospital Peking University Shenzhen China

**Keywords:** people living with HIV and AIDS, COVID-19 vaccination, willingness, perceptions, internet and social media influences, interpersonal communication

## Abstract

**Background:**

HIV infection is a significant independent risk factor for both severe COVID-19 presentation at hospital admission and in-hospital mortality. Available information has suggested that people living with HIV and AIDS (PLWHA) could benefit from COVID-19 vaccination. However, there is a dearth of evidence on willingness to receive COVID-19 vaccination among PLWHA.

**Objective:**

The aim of this study was to investigate willingness to receive COVID-19 vaccination among a national sample of PLWHA in China.

**Methods:**

This cross-sectional online survey investigated factors associated with willingness to receive COVID-19 vaccination among PLWHA aged 18 to 65 years living in eight conveniently selected Chinese metropolitan cities between January and February 2021. Eight community-based organizations (CBOs) providing services to PLWHA facilitated the recruitment. Eligible PLWHA completed an online survey developed using a widely used encrypted web-based survey platform in China. We fitted a single logistic regression model to obtain adjusted odds ratios (aORs), which involved one of the independent variables of interest and all significant background variables. Path analysis was also used in the data analysis.

**Results:**

Out of 10,845 PLWHA approached by the CBOs, 2740 completed the survey, and 170 had received at least one dose of the COVID-19 vaccine. This analysis was performed among 2570 participants who had never received COVID-19 vaccination. Over half of the participants reported willingness to receive COVID-19 vaccination (1470/2570, 57.2%). Perceptions related to COVID-19 vaccination were significantly associated with willingness to receive COVID-19 vaccination, including positive attitudes (aOR 1.11, 95% CI 1.09-1.12; *P*<.001), negative attitudes (aOR 0.96, 95% CI 0.94-0.97; *P*<.001), perceived support from significant others (perceived subjective norm; aOR 1.53, 95% CI 1.46-1.61; *P*<.001), and perceived behavioral control (aOR 1.13, 95% CI 1.11-1.14; *P*<.001). At the interpersonal level, receiving advice supportive of COVID-19 vaccination from doctors (aOR 1.99, 95% CI 1.65-2.40; *P*<.001), CBO staff (aOR 1.89, 95% CI 1.51-2.36; *P*<.001), friends and/or family members (aOR 3.22, 95% CI 1.93-5.35; *P*<.001), and PLWHA peers (aOR 2.38, 95% CI 1.85-3.08; *P*<.001) was associated with higher willingness to receive COVID-19 vaccination. The overall opinion supporting COVID-19 vaccination for PLWHA on the internet or social media was also positively associated with willingness to receive COVID-19 vaccination (aOR 1.59, 95% CI 1.31-1.94; *P*<.001). Path analysis indicated that interpersonal-level variables were indirectly associated with willingness to receive COVID-19 vaccination through perceptions (β=.43, 95% CI .37-.51; *P*<.001).

**Conclusions:**

As compared to PLWHA in other countries and the general population in most parts of the world, PLWHA in China reported a relatively low willingness to receive COVID-19 vaccination. The internet and social media as well as interpersonal communications may be major sources of influence on PLWHA’s perceptions and willingness to receive COVID-19 vaccination.

## Introduction

The World Health Organization (WHO) confirmed that HIV infection is a significant independent risk factor for both severe COVID-19 presentation at hospital admission and in-hospital mortality [1]. It is essential to take additional measures to prevent people living with HIV and AIDS (PLWHA) from contracting COVID-19.

The COVID-19 vaccine offers the best hope for ending the pandemic. Simulation experiments showed that when vaccine efficacy was 80%, 75% coverage could end the COVID-19 pandemic without any other control measures [2]. A relatively small number of PLWHA were involved in phase III COVID-19 vaccine trials. The Pfizer study and the Oxford/AstraZeneca study recruited 196 and 157 PLWHA, respectively; however, the data on vaccine efficacy for PLWHA was not included in the publications that led to their approval in the United States and the United Kingdom [3,4]. The Moderna study included 176 PLWHA [5]. Of the PLWHA in the Moderna study, one person who received the placebo and none who received the vaccine developed COVID-19. The Janssen (Johnson & Johnson) study included 1218 PLWHA [6]. Two PLWHA who received the vaccine and four who received the placebo developed COVID-19. There were 201 PLWHA in the Novavax study; the overall vaccine efficacy was 49.4%, with a higher efficacy when PLWHA were excluded from the analysis (60%) [7]. A number of studies observed similar immune responses and adverse events in response to messenger RNA (mRNA) and adenovirus vector COVID-19 vaccines between PLWHA and HIV-negative individuals [8-12]. Despite limited evidence, available information suggests that COVID-19 vaccines recommended by the WHO are safe for PLWHA. There is no evidence to support a less robust response to COVID-19 vaccines among PLWHA. PLWHA could benefit from COVID-19 vaccination.

The recommendations or guidelines regarding COVID-19 vaccination for PLWHA are inconsistent across countries. The WHO, the United States Department of Health and Human Services, the British HIV Association, and health authorities in Australia recommend that PLWHA receive COVID-19 vaccination regardless of their CD4+ T cell counts [1,13-15]. PLWHA comprise one of the priority groups to receive COVID-19 vaccination in the United Kingdom, the United States, and Australia [13-15]. Moreover, the United States Centers for Disease Control and Prevention recommends that PLWHA who are moderately to severely immunocompromised should receive an additional dose of mRNA COVID-19 vaccine after the initial doses [16]. In the Asia-Pacific region, Singapore used to recommend COVID-19 vaccination to PLWHA who are receiving antiretroviral therapy (ART), with suppressed HIV viral load, and with CD4+ T cell counts over 200 cells/µL [17]. Their recommendation expanded to all PLWHA regardless of ART, viral suppression, or CD4+ T cell counts since July 2021 [17]. At the time when this study was conducted, immunodeficiency, including HIV infection, was listed as a precaution for COVID-19 vaccination in China; PLWHA were asked to seek advice from doctors regarding COVID-19 vaccination [18]. The guideline was updated one month after the completion of this study (March 2021) and recommended COVID-19 vaccination for PLWHA, regardless of their CD4+ T cell counts [18].

Vaccine hesitancy hindered the successful control of the COVID-19 pandemic. Therefore, it is helpful for governments to plan interventions to improve people’s awareness of the safety and benefits of the COVID-19 vaccine and to reduce vaccine hesitancy. In order to promote the COVID-19 vaccination of PLWHA, it is necessary to understand their willingness to receive COVID-19 vaccination and related facilitators and barriers. However, most studies investigating willingness to receive COVID-19 vaccination and its associated factors were conducted among the general population and medical professionals [19-21]; therefore, the findings might not be applicable to PLWHA. To our knowledge, only two published studies investigated COVID-19 vaccine hesitancy among PLWHA in the United States and France [22,23]. The results showed that 28.7% of PLWHA in France declared hesitancy to be vaccinated against COVID-19 [22]. Over 30% of PLWHA in the United States indicated that if a vaccine was available to prevent COVID-19, they would not trust it (34%) nor want to get it (32%) [23]. Concerns about their health and the belief that COVID-19 vaccination should be mandatory and is important for people with chronic disease were associated with higher willingness to receive COVID-19 vaccination among PLWHA, while a previous history of vaccination refusal, mistrust in public health information, and concerns related to side effects were shown to be barriers [22,23].

We applied the socioecological model to understand factors associated with willingness to receive COVID-19 vaccination among PLWHA at individual, interpersonal, and sociostructural levels [24]. Interventions addressing influencing factors at multiple levels are more likely to be successful [24]. The socioecological model was used successfully to explain compliance to COVID-19 personal preventive measures among Chinese populations [25]. At the sociostructural level, two COVID-19 vaccination delivery models were implemented simultaneously in China at the time of this study. Individuals could make an appointment to receive COVID-19 vaccination in some cities, while COVID-19 vaccination was mainly arranged by employers and did not allow individuals to make appointments in other Chinese cities. People had the right to refuse such an arrangement. As of the writing of this paper, since the number of vaccines is inadequate to cover the entire Chinese population at the initial phase, priority is given to subgroups with elevated risks of developing COVID-19 (eg, health care workers, pandemic-control staff, and cold-chain workers). Some Chinese cities also reported a shortage of COVID-19 vaccines. We expected that these sociostructural-level factors would influence PLWHA’s willingness to receive COVID-19 vaccination. At the individual level, perceived efficacy, concerns about side effects, others’ acceptance, and confidence to receive the vaccine influenced people’s willingness to receive COVID-19 vaccination [26-30]. At the interpersonal level, people were exposed to information related to COVID-19 vaccination through interpersonal communication or the internet and social media. Higher exposure to positive information related to COVID-19 vaccination on social media was associated with a higher willingness to receive such vaccination among Chinese factory workers [27]. Interpersonal communication, such as receiving advice from doctors and family members, was also positively associated with willingness to receive COVID-19 vaccination among the general population in China [28]. Clinical doctors and staff from community-based organizations (CBOs) are the main service providers for PLWHA [31]. Their advice regarding COVID-19 vaccination may have a great impact on PLWHA’s decisions to accept such vaccination. A recent study suggested that exposure to positive information related to COVID-19 vaccination increased perceptions favoring such vaccination [27]. In this study, we hypothesized that exposure to information supporting PLWHA in receiving COVID-19 vaccination through the internet and social media as well as interpersonal communication would influence PLWHA’s perceptions of such vaccination and, hence, affect their willingness to receive COVID-19 vaccination.

To our knowledge, no studies have investigated willingness to receive COVID-19 vaccination among PLWHA in China. To address knowledge gaps, this study investigated willingness to receive COVID-19 vaccination among a national sample of PLWHA. We examined the effects of the following factors: sociodemographics, HIV-related characteristics, individual-level factors (ie, perceptions related to COVID-19 vaccination), interpersonal-level variables (ie, exposure to COVID-19 vaccination–related information through the internet and social media as well as interpersonal communication), and sociostructural-level factors (ie, COVID-19 vaccination delivery model, members of priority groups, and shortage in vaccine supply). We further tested the hypothesis that perceptions of COVID-19 vaccination would mediate the association between interpersonal-level variables and willingness to receive COVID-19 vaccination.

## Methods

### Study Design

**Figure 1:**
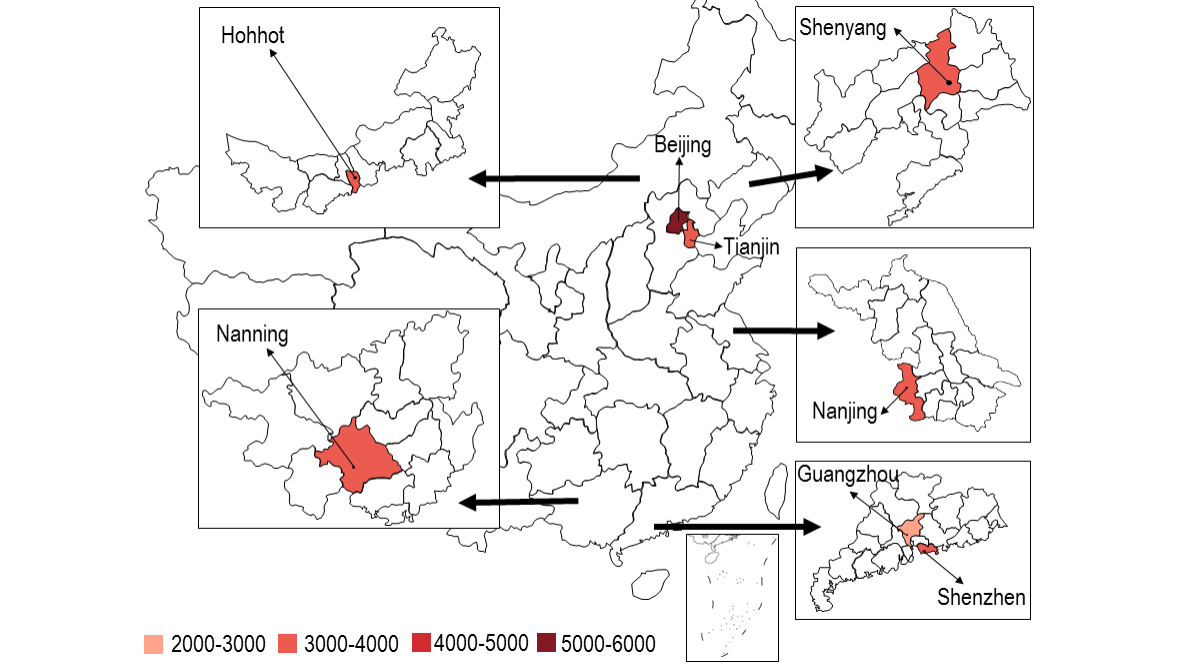
Number of patients living with HIV and AIDS in different study sites.

**Figure 2:**
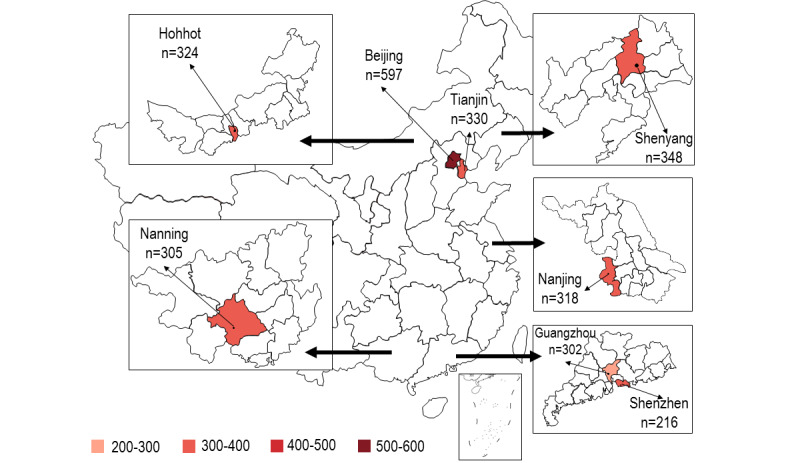
Number of participants in different study sites.

**Figure 3:**
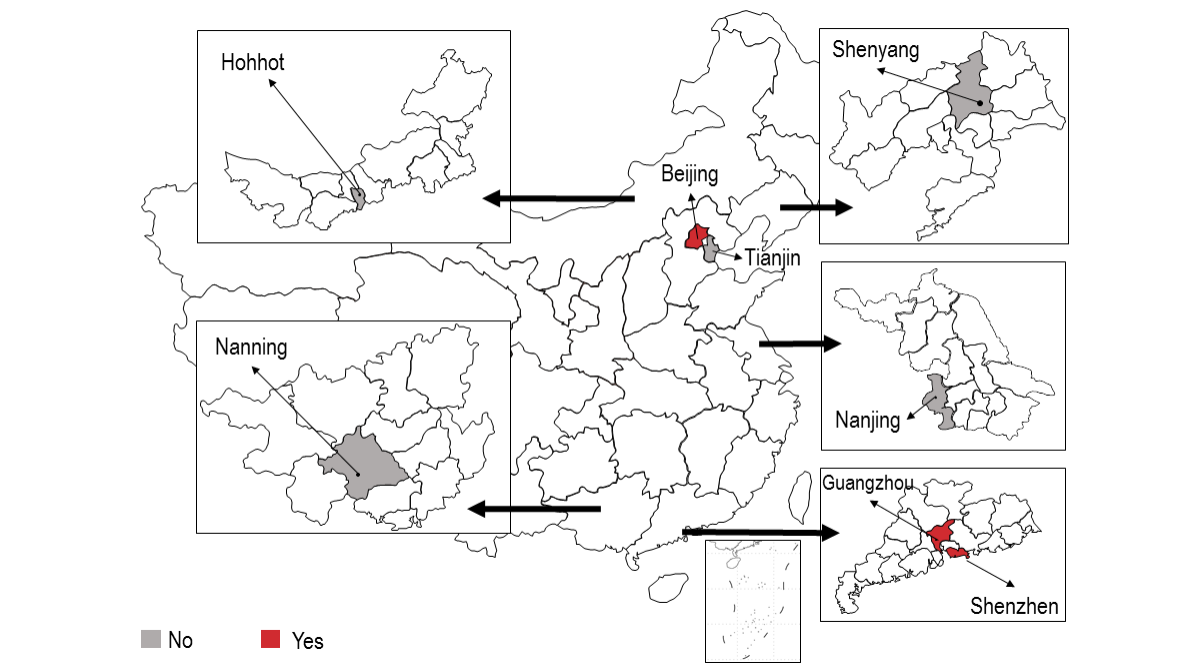
Participants' ability ("Yes") or inability ("No") to make appointments to receive COVID-19 vaccination in different study sites.

This study makes use of a multicenter cross-sectional online survey conducted in eight conveniently selected large Chinese cities between January and February 2021. These cities included two in the north (Tianjin and Beijing), two in the northeast (Shenyang and Hohhot), one in the east (Nanjing), and three in the south (Nanning, Guangzhou, and Shenzhen). Beijing is the capital city of China. Shenzhen is a major special economic zone in China bordering Hong Kong in the south. The other six cities are capital cities of the provinces. Reasons for selecting these cities included the following: (1) each city has a CBO providing services to PLWHA, (2) each city has a large number of PLWHA, and (3) COVID-19 vaccination was first scaled up at these sites. At the time of this study, people in Beijing, Guangzhou, and Shenzhen could make an appointment to receive COVID-19 vaccination. The procedures of making an appointment were simple. People first downloaded a smartphone app developed by the health bureau. After logging in, they could choose the time and location to receive COVID-19 vaccination. In the other five cities, vaccination was arranged by employers and did not allow individuals to make appointments. Only two types of inactivated COVID-19 vaccines—Sinovac-CoronaVac and Sinopharm—were available in China during the study period. They were provided by designated community vaccination centers and people could only receive them at sites in these centers. Immunodeficiency, including HIV infection, was listed as a precaution for COVID-19 vaccination in China during the study period; it was recommended that PLWHA seek advice from doctors regarding COVID-19 vaccination [18]. Participants who had never received COVID-19 vaccination were asked about their willingness to receive the vaccination. The context of this study is shown in Figures 1 to 3 .

### Study Population

Study participants included individuals aged 18 to 65 years who have been diagnosed with HIV or AIDS and were living in one of the eight cities. We did not include PLWHA older than 65 years, as COVID-19 vaccination was not approved for this age group in China at the time of the survey. Exclusion criteria included the following: (1) being illiterate and unable to complete the questionnaire survey and (2) being ineligible for COVID-19 vaccination (eg, pregnancy, latency, and severe allergy to previous vaccination).

### Recruitment and Data Collection

The eight CBOs that were mainly providing services to marginalized populations (eg, PLWHA and HIV high-risk populations), one in each study site, facilitated recruitment through their networks. These CBOs have been working closely with HIV clinical service providers. CBOs in China are the main providers of HIV outreach services to PLWHA, as these routine tasks have been transferred from government agencies to CBOs [31]. A high proportion of PLWHA are followed up by the CBOs. WeChat is the most common live-chat app used by CBOs to connect with PLWHA clients. The research team provided training for CBO staff who were responsible for communications with PLWHA within the scope of their routine service. Participants were recruited by posting advertisements in the WeChat groups involving PLWHA clients kept by the CBOs. The advertisements contained study information and contacts of project staff (ie, private WeChat account number and telephone number). Interested participants were asked to contact CBO staff either using private WeChat messages or telephone calls. CBO staff screened prospective participants using the eligibility criteria, introduced the study purpose and procedures, answered questions, and explained the confidentiality of study participation. Participation in this study was voluntary, and participants could refuse to answer any of the questions and withdraw from the study at any time without consequences. Participants signed an electronic consent form sent via WeChat messages. A link to access an online self-administered questionnaire was sent to the consented participants.

The survey was carried out through Golden Data, a commonly used, encrypted, web-based survey platform in China. Each individual WeChat account was allowed to access the online questionnaire only once to avoid duplicate responses. The Golden Data tool performed a completeness check before each questionnaire was submitted. Participants could review and change their responses when they completed the questionnaire. The survey took about 13 to 15 minutes to complete. An electronic coupon with a value of 20 Chinese yuan (US $3.1) was sent to each participant upon the completion of the survey. A unique ID was assigned to each participant, which was used to delink the study database from personal identifying data. All data collected by the online surveys were stored in the Golden Data server and protected by a password. Only the designated research team members had access to the database.

### Ethics Approval and Consent to Participate

Signed electronic informed consent forms were obtained from all subjects involved in the study. These were kept separately from the empirical data and stored in a password-protected computer or a locked cabinet in the same locked office. This study was conducted according to the guidelines of the Declaration of Helsinki and approved by the Institutional Review Boards of Changzhi Medical College (protocol code RT2021003).

### Measurements

#### Development of the Questionnaire

A panel consisting of public health researchers, health psychologists, clinicians, CBO staff, and PLWHA was formed to develop the questionnaire used in this study. The panel revised and finalized the questionnaire based on a pilot test among 10 PLWHA. These 10 PLWHA did not participate in the actual survey.

#### Background Characteristics

Participants reported sociodemographic characteristics, lifestyles (ie, smoking and alcohol drinking), height and weight, and history of other vaccinations in the past 3 years. Participants were also asked whether they had any chronic conditions, such as chronic cardiovascular, respiratory, kidney, and liver diseases; hypertension; diabetes mellitus and its chronic complications; cancers; lymphoma; leukemia; autoimmune diseases; hemorrhagic diseases; and history of severe allergy. The survey also collected some characteristics related to HIV infection (eg, time since HIV diagnosis, whether they were receiving ART, HIV viral load and CD4+ T cell count in the most recent episode of testing, and self-reported severity of AIDS-related symptoms).

#### Willingness to Receive COVID-19 Vaccination

Participants were asked about their likelihood of receiving free COVID-19 vaccination in the future; the response categories were as follows: 1 (very unlikely), 2 (unlikely), 3 (neutral), 4 (likely), and 5 (very likely). This study defined willingness to receive COVID-19 vaccination as the responses “likely” or “very likely” [27].

#### Variables at the Sociostructural, Individual, and Interpersonal Levels Related to COVID-19 Vaccination

The research team interviewed staff from the Chinese Center for Disease Control and Prevention who were responsible for implementing the COVID-19 vaccination program about whether individuals were allowed to make an appointment to receive COVID-19 vaccination and whether there was a shortage of COVID-19 vaccines during the project period at different study sites. Participants were asked whether they belonged to any of the groups who had priority to receive COVID-19 vaccination as listed by the National Health Commission during the project period.

At the individual level, four scales were constructed to measure perceptions related to COVID-19 vaccination. These scales were as follows: (1) the 5-item Positive Attitude Scale, (2) the 5-item Negative Attitude Scale, (3) the 4-item Perceived Subjective Norm Scale (ie, whether significant others would support them to receive COVID-19 vaccination), and (4) the 5-item Perceived Behavioral Control Scale (ie, how much control PLWHA have for receiving COVID-19 vaccination). Response categories were as follows: 1 (strongly disagree), 2 (disagree), 3 (neutral), 4 (agree), and 5 (strongly agree). Positive and negative attitudes, perceived subjective norm, and perceived behavioral control were significantly associated with willingness to receive COVID-19 vaccination among Chinese people [27].

For interpersonal-level variables, participants were asked whether they received advice given by doctors, CBO staff, friends and/or family members, and other PLWHA regarding COVID-19 vaccination. Participants were also asked about the overall opinion regarding COVID-19 vaccination that they found on the internet or social media; response categories were as follows: 1 (against taking up COVID-19 vaccination), 2 (no advice/neutral), and 3 (supportive in taking up COVID-19 vaccination).

### Statistical Analysis

Using willingness to receive COVID-19 vaccination as the dependent variable and background characteristics as independent variables, crude odds ratios were obtained by logistic regression models. After adjusting for variables with *P*<.05 in the univariate analysis, associations between independent variables of interest (ie, individual-, interpersonal-, and sociostructural-level variables) and the dependent variable were then assessed by adjusted odds ratios (aORs) and 95% CIs. Each aOR was obtained by fitting a single logistic regression model, which involved one of the independent variables of interest and all significant background characteristics.

Path analysis was conducted to test the mediation model. The mean scores of the Positive Attitude Scale, the Negative Attitude Scale, the Perceived Subjective Norm Scale, and the Perceived Behavioral Control Scale were used as indicators to represent the latent variable of perceptions related to COVID-19 vaccination. The mean scores of advice given by doctors, CBO staff, friends and/or family members, and other PLWHA, as well as the overall opinion on the internet and social media, were used as indicators to represent the latent variable of interpersonal-level variables. Confirmatory factor analysis was conducted to test goodness of fit of these constructs. The latent variable representing perceptions was used as the independent variable, and willingness to receive COVID-19 vaccination was used as the dependent variable. The significant background characteristics were controlled for the model. Goodness of fit was tested using chi-square tests, the comparative fit index (CFI), the Tucker-Lewis index (TLI), and the root mean square error of approximation (RMSEA). Standardized path coefficients (β) and unstandardized path coefficients (B) were reported. The asymmetric CIs based on the bootstrap method (10,000 times) that were used for significance testing of mediation hypotheses with the 95% bootstrap CI did not include zero, indicating a statistically significant mediation effect. The level of statistical significance was set at *P*<.05. SPSS for Windows (version 21.0; IBM Corp) and Mplus (version 8.3; Muthén and Muthén) were used for all analyses.

## Results

### Background Characteristics

**Figure 4:**
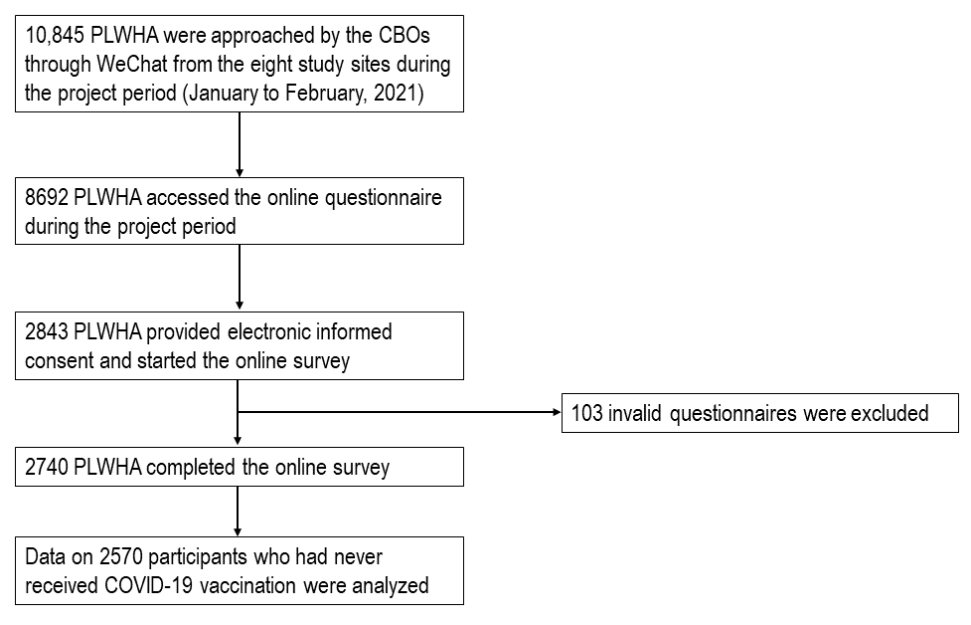
Flowchart of data collection. CBO: community-based organization; PLWHA: people living with HIV and AIDS.

The CBOs approached 10,845 PLWHA in their WeChat groups, 8692 accessed the online survey, 2740 completed the survey, and 170 received at least one dose of COVID-19 vaccination at the time of the survey. This study was based on 2570 eligible participants who had never received COVID-19 vaccination; the flowchart of this study is presented in Figure 4.

The majority of the 2570 participants were 18 to 39 years of age (n=1916, 74.6%), identified themselves as male (n=2106, 81.9%), were currently single (n=1750, 68.1%), had a full-time job (n=1782, 69.3%), and only had basic health insurance (n=1927, 75.0%). Regarding characteristics related to HIV infection, 17.2% (n=443) of the 2570 participants received their diagnosis within 1 year, 97.3% (n=2501) were receiving ART, 67.9% (n=1746) reported undetectable viral load, and 46.0% (n=1181) reported their CD4+ T cell count level to be above 500/µL. Less than 5% of the participants self-reported having severe AIDS-related symptoms (Tables 1 and 2).

**Table 1:** Sociodemographic characteristics of 2570 unvaccinated people living with HIV and AIDS in eight Chinese cities.

Characteristic		All participants (N=2570), n (%)	Participants willing to receive COVID-19 vaccination (n=1470), n (%)	Participants unwilling to receive COVID-19 vaccination (n=1100), n (%)	Participants willing versus unwilling, cOR^a^ (95% CI)	*P* value
**Age group (years)**						
	18-29	791 (30.8)	456 (31.0)	335 (30.5)	1.0	Ref^b^
	30-39	1125 (43.8)	638 (43.4)	487 (44.3)	0.96 (0.80-1.16)	.68
	40-49	475 (18.5)	287 (19.5)	188 (17.1)	1.12 (0.89-1.41)	.33
	≥50	179 (7.0)	89 (6.1)	90 (8.2)	0.73 (0.53-1.01)	.054
**Gender at birth**						
	Male	2431 (94.6)	1390 (94.6)	1041 (94.6)	1.0	Ref
	Female	139 (5.4)	80 (5.4)	59 (5.4)	1.02 (0.72-1.44)	.93
**Gender identity**						
	Male	2106 (81.9)	1224 (83.3)	882 (80.2)	1.0	Ref
	Female	228 (8.9)	127 (8.6)	101 (9.2)	0.91 (0.69-1.19)	.48
	Transgender	228 (8.9)	114 (7.8)	114 (10.4)	0.72 (0.55-0.95)	.02
	Others	8 (0.3)	5 (0.3)	3 (0.3)	1.20 (0.29-5.04)	.80
**Relationship status**						
	Currently single	1750 (68.1)	993 (67.6)	757 (68.8)	1.0	Ref
	Cohabitating or married with a same-sex partner	377 (14.7)	217 (14.8)	160 (14.5)	1.03 (0.83-1.30)	.77
	Cohabitating or married with an opposite-sex partner	443 (17.2)	260 (17.7)	183 (16.6)	1.08 (0.88-1.34)	.46
**Highest education level attained**						
	Junior high school or below	425 (16.5)	249 (16.9)	176 (16.0)	1.0	Ref
	Senior high school or equivalent	574 (22.3)	319 (21.7)	255 (23.2)	0.88 (0.69-1.14)	.34
	College and above	1571 (61.1)	902 (61.4)	669 (60.8)	0.95 (0.77-1.18)	.66
**Employment status**						
	Full time	1782 (69.3)	1022 (69.5)	760 (69.1)	1.0	Ref
	Part time, unemployed, retired, student, or others	788 (30.7)	448 (30.5)	340 (30.9)	0.98 (0.83-1.16)	.81
**Monthly personal income (Chinese yuan^c^)**						
	No fixed income	302 (11.8)	174 (11.8)	128 (11.8)	1.0	Ref
	<1000	136 (5.3)	72 (4.9)	64 (5.8)	0.83 (0.55-1.24)	.36
	1000-2999	338 (13.2)	199 (13.5)	139 (12.6)	1.05 (0.77-1.44)	.75
	3000-4999	736 (28.6)	413 (28.1)	323 (29.4)	0.94 (0.72-1.23)	.66
	5000-6999	492 (19.1)	285 (19.4)	207 (18.8)	1.01 (0.76-1.35)	.93
	7000-9999	273 (10.6)	174 (11.8)	99 (9.0)	1.29 (0.92-1.81)	.13
	≥10,000	293 (11.4)	153 (10.4)	140 (12.7)	0.80 (0.58-1.11)	.19
**Type of health insurance**						
	None	307 (11.9)	166 (11.3)	141 (12.8)	1.0	Ref
	Basic health insurance only	1927 (75.0)	1111 (75.6)	816 (74.2)	1.16 (0.91-1.47)	.24
	Commercial health insurance only	69 (2.7)	47 (3.2)	22 (2.0)	1.82 (1.04-3.16)	.04
	Both basic and commercial health insurance	253 (9.8)	140 (9.5)	113 (10.3)	1.05 (0.75-1.47)	.77
	Others	14 (0.5)	6 (0.4)	8 (0.7)	0.64 (0.22-1.88)	.41

^a^cOR: crude odds ratio.

^b^Ref: reference.

^c^A currency exchange rate of 1 Chinese yuan=US $0.16 is applicable.

**Table 2:** Lifestyle and health conditions of 2570 unvaccinated people living with HIV and AIDS in eight Chinese cities.

Condition			All participants (N=2570), n (%)		Participants willing to receive COVID-19 vaccination (n=1470), n (%)		Participants unwilling to receive COVID-19 vaccination (n=1100), n (%)		Participants willing versus unwilling, cOR^a^ (95% CI)		*P* value
**Current smoker**											
	No	1855 (72.2)		1069 (72.7)		786 (71.5)		1.0		Ref^b^	
	Yes	715 (27.8)		401 (27.3)		314 (28.5)		0.94 (0.79-1.12)		.48	
**Current drinker**											
	No	2068 (80.5)		1190 (81.0)		878 (79.8)		1.0		Ref	
	Yes	502 (19.5)		280 (19.0)		222 (20.2)		0.93 (0.77-1.13)		.47	
**Self-reported BMI (kg/m^2^)**											
	<18.5	235 (9.1)		125 (8.5)		110 (10.0)		1.0		Ref	
	18.5-23.9	1649 (64.2)		944 (64.2)		705 (64.1)		1.18 (0.90-1.55)		.24	
	24.0-27.9	558 (21.7)		327 (22.2)		231 (21.0)		1.25 (0.92-1.69)		.16	
	≥28	127 (4.9)		74 (5.0)		53 (4.8)		1.23 (0.80-1.90)		.36	
**Presence of chronic disease conditions**											
	No	1707 (66.4)		1009 (68.6)		698 (63.5)		1.0		Ref	
	Yes	863 (33.6)		461 (31.4)		402 (36.5)		0.79 (0.67-0.94)		.01	
**Medication use for treating chronic diseases**											
	No	2411 (93.8)		1385 (94.2)		1026 (93.3)		1.0		Ref	
	Yes	159 (6.2)		85 (5.8)		74 (6.7)		0.85 (0.62-1.17)		.33	
**History of other vaccinations in the past 3 years**											
	No	2002 (77.9)		1110 (75.5)		892 (81.1)		1.0		Ref	
	Yes	568 (22.1)		360 (24.5)		208 (18.9)		1.39 (1.15-1.69)		.001	
**Time since HIV diagnosis (years)**											
	≤1	443 (17.2)		264 (18.0)		179 (16.3)		1.0		Ref	
	2-5	1198 (46.6)		685 (46.6)		513 (46.6)		0.91 (0.73-1.13)		.38	
	>5	929 (36.1)		521 (35.4)		408 (37.1)		0.87 (0.69-1.09)		.22	
**Receiving antiretroviral therapy**											
	No	69 (2.7)		40 (2.7)		29 (2.6)		1.0		Ref	
	Yes	2501 (97.3)		1430 (97.3)		1071 (97.4)		0.97 (0.60-1.57)		.90	
**HIV viral load in the most recent episode of testing (copies/mL)**											
	Undetectable (<50)	1746 (67.9)		988 (67.2)		758 (68.9)		1.0		Ref	
	50-200	154 (6.0)		79 (5.4)		75 (6.8)		0.81 (0.58-1.12)		.21	
	201-400	69 (2.7)		45 (3.1)		24 (2.2)		1.44 (0.87-2.38)		.16	
	>400	137 (5.3)		87 (5.9)		50 (4.5)		1.34 (0.93-1.91)		.12	
	Not sure	464 (18.1)		271 (18.4)		193 (17.5)		1.08 (0.88-1.33)		.48	
**CD4+ T cell count in the most recent episode of testing (cells/mm^3^)**											
	≥500	1181 (46.0)		669 (45.5)		512 (46.5)		1.0		Ref	
	350-499	531 (20.7)		320 (21.8)		211 (19.2)		1.16 (0.94-1.43)		.16	
	200-349	258 (10.0)		150 (10.2)		108 (9.8)		1.06 (0.81-1.40)		.66	
	<200	89 (3.5)		44 (3.0)		45 (4.1)		0.75 (0.49-1.15)		.19	
	Unknown	511 (19.9)		287 (19.5)		224 (20.4)		0.98 (0.80-1.21)		.85	
**Self-reported severity of AIDS-related symptoms**											
	No symptoms	1306 (50.8)		767 (52.2)		539 (49.0)		1.0		Ref	
	Mild	839 (32.6)		483 (32.9)		356 (32.4)		0.95 (0.80-1.14)		.60	
	Moderate	308 (12.0)		157 (10.7)		151 (13.7)		0.73 (0.57-0.94)		.01	
	Severe	117 (4.6)		63 (4.3)		54 (4.9)		0.82 (0.56-1.20)		.31	

^a^cOR: crude odds ratio.

^b^Ref: reference.

### Willingness to Receive COVID-19 Vaccination and Variables at the Individual, Interpersonal, and Sociostructural Levels

Over half of the participants were willing to receive free COVID-19 vaccinations in the future (1470/2570, 57.2%). A shortage of COVID-19 vaccines was encountered in Shenyang, Guangzhou, and Shenzhen. Among the participants, 19.0% (488/2570) identified themselves as belonging to a priority group that would receive COVID-19 vaccination. The Cronbach α of the scales for perceptions related to COVID-19 vaccination ranged from .83 to .92; single factors were identified by exploratory factor analysis, explaining 61.1% to 76.4% of the total variance (Table 3 and Multimedia Appendix 1).

**Table 3:** Willingness to receive COVID-19 vaccination and variables at the sociostructural, individual, and interpersonal levels among 2570 unvaccinated people living with HIV and AIDS (PLWHA).

Variable			All participants (N=2570)	Participants willing to receive COVID-19 vaccination (n=1470)	Participants unwilling to receive COVID-19 vaccination (n=1100)	Participants willing versus unwilling, cOR^a^ (95% CI)	*P* value
**Willing to receive free COVID-19 vaccination, n (%)**							
	No (very unlikely, unlikely, or neutral)		1100 (42.8)	0 (0)	1100 (100)	N/A^b^	N/A
	Yes (likely or very likely)		1470 (57.2)	1470 (100)	0 (0)	N/A	N/A
**Sociostructural-level variables, n (%)**							
	**Individuals could make an appointment to receive COVID-19 vaccination during the study period**						
		No	1578 (61.4)	887 (60.3)	691 (62.8)	1.0	Ref^c^
		Yes	992 (38.6)	583 (39.7)	409 (37.2)	1.11 (0.95-1.30)	.20
	**There was a shortage of COVID-19 vaccine in the city where the participants were living during the study period**						
		No	1729 (67.3)	1009 (68.6)	720 (65.5)	1.0	Ref
		Yes	841 (32.7)	461 (31.4)	380 (34.5)	0.87 (0.73-1.02)	.09
	**Participants belonged to priority groups that would receive COVID-19 vaccination in their cities during the study period**						
		No	2082 (81.0)	1189 (80.9)	893 (81.2)	1.0	Ref
		Yes	488 (19.0)	281 (19.1)	207 (18.8)	1.02 (0.84-1.25)	.85
**Individual-level variables: perceptions and attitudes toward COVID-19 vaccination, mean (SD)**							
	Positive Attitude Scale^d^ score		18.4 (4.8)	19.3 (4.6)	17.1 (4.7)	1.11 (1.09-1.13)	<.001
	Negative Attitude Scale^e^ score		18.6 (5.2)	18.1 (5.4)	19.3 (4.8)	0.96 (0.94-0.97)	<.001
	Perceived Subjective Norm Scale^f^ score		13.3 (2.4)	14.5 (2.5)	12.3 (1.8)	1.53 (1.46-1.61)	<.001
	Perceived Behavioral Control Scale^g^ score		12.9 (6.1)	14.7 (6.1)	10.6 (5.4)	1.13 (1.11-1.14)	<.001
**Interpersonal-level variables, mean (SD)**							
	Advice from doctors regarding COVID-19 vaccination		2.1 (0.5)	2.2 (0.5)	2.0 (0.4)	2.03 (1.69-2.44)	<.001
	Advice from CBO^h^ staff regarding COVID-19 vaccination		2.1 (0.4)	2.1 (0.4)	2.0 (0.4)	1.86 (1.49-2.32)	<.001
	Advice from friends and/or family members regarding COVID-19 vaccination		2.0 (0.2)	2.0 (0.4)	1.9 (0.2)	3.18 (1.92-5.26)	<.001
	Advice from other PLWHA regarding COVID-19 vaccination		2.0 (0.3)	2.1 (0.3)	1.9 (0.4)	2.38 (1.85-3.07)	<.001
	Overall opinion regarding COVID-19 vaccination for PLWHA on the internet and social media		2.0 (0.4)	2.1 (0.4)	2.0 (0.4)	1.63 (1.34-1.98)	<.001

^a^cOR: crude odds ratio.

^b^N/A: not applicable; cOR was not calculated for this item.

^c^Ref: reference.

^d^The Positive Attitude Scale includes five items and has a maximum score of 25; Cronbach α=.83; one factor was identified by exploratory factor analysis, explaining 61.1% of the total variance.

^e^The Negative Attitude Scale includes five items and has a maximum score of 25; Cronbach α=.87; one factor was identified by exploratory factor analysis, explaining 66.3% of the total variance.

^f^The Perceived Subjective Norm Scale includes four items and has a maximum score of 20; Cronbach α=.84; one factor was identified by exploratory factor analysis, explaining 63.4% of the total variance.

^g^The Perceived Behavioral Control Scale includes five items and has a maximum score of 25; Cronbach α=.92; one factor was identified by exploratory factor analysis, explaining 76.4% of the total variance.

^h^CBO: community-based organization.

### Factors Associated With Willingness to Receive COVID-19 Vaccination

In the univariate logistic regression analysis, transgender persons, those with chronic conditions, and those with more severe AIDS-related symptoms showed lower willingness to receive COVID-19 vaccination. Having commercial health insurance only and a history of other vaccinations in the past 3 years were associated with higher willingness to receive COVID-19 vaccination (Tables 1 and 2).

After adjusting for significant background characteristics, having more positive attitudes toward COVID-19 vaccination (aOR 1.11, 95% CI 1.09-1.12; *P*<.001), stronger perceived support from significant others (perceived subjective norm; aOR 1.53, 95% CI 1.46-1.61; *P*<.001), and higher perceived behavioral control (aOR 1.13, 95% CI 1.11-1.14; *P*<.001) to take up the vaccination were associated with higher willingness to receive COVID-19 vaccination. A negative association was found between negative attitudes toward COVID-19 vaccination and the dependent variable (aOR 0.96, 95% CI 0.94-0.97; *P*<.001). At the interpersonal level, receiving advice supportive of COVID-19 vaccination from doctors (aOR 1.99, 95% CI 1.65-2.40; *P*<.001), CBO staff (aOR 1.89, 95% CI 1.51-2.36; *P*<.001), friends and/or family members (aOR 3.22, 95% CI 1.93-5.35; *P*<.001), and other PLWHA (aOR 2.38, 95% CI 1.85-3.08; *P*<.001) were associated with higher willingness to receive COVID-19 vaccination. The overall opinion supporting COVID-19 vaccination for PLWHA on the internet and social media was also positively associated with the dependent variable (aOR 1.59, 95% CI 1.31-1.94; *P*<.001; Table 4).

**Table 4:** Factors associated with willingness to receive COVID-19 vaccination among 2570 unvaccinated people living with HIV and AIDS (PLWHA).

Variable				aOR^a^ (95% CI)		*P* value
**Sociostructural-level variables**						
	**Individuals could make an appointment to receive COVID-19 vaccination during the study period**					
		No	N/A^b^		N/A	
		Yes	N/A		N/A	
	**There was a shortage of COVID-19 vaccines in the city where the participants were living during the study period**					
		No	N/A^b^		N/A	
		Yes	N/A		N/A	
	**Participants belonged to priority groups that would receive COVID-19 vaccination in their cities during the study period**					
		No	N/A^b^		N/A	
		Yes	N/A		N/A	
**Individual-level variables**						
	Positive Attitude Scale score			1.11 (1.09-1.12)		<.001
	Negative Attitude Scale score			0.96 (0.94-0.97)		<.001
	Perceived Subjective Norm Scale score			1.53 (1.46-1.61)		<.001
	Perceived Behavioral Control Scale score			1.13 (1.11-1.14)		<.001
**Interpersonal-level variables**						
	Advice from doctors regarding COVID-19 vaccination			1.99 (1.65-2.40)		<.001
	Advice from CBO^c^ staff regarding COVID-19 vaccination			1.89 (1.51-2.36)		<.001
	Advice from friends and/or family members regarding COVID-19 vaccination			3.22 (1.93-5.35)		<.001
	Advice from other PLWHA regarding COVID-19 vaccination			2.38 (1.85-3.08)		<.001
	Overall opinion regarding COVID-19 vaccination for PLWHA on the internet and social media			1.59 (1.31-1.94)		<.001

^a^aOR: adjusted odds ratio; odds ratios were obtained by fitting a single logistic regression model involving an independent variable of interest and all background variables listed in Tables 1 and 2 with *P*<.05 in univariate analysis.

^b^Univariate analyses of these variables yielded *P*>.05, so multivariate analyses were not conducted.

^c^CBO: community-based organization.

### Testing the Mediation Effects of Perceptions on the Association Between Interpersonal-Level Variables and Willingness to Receive COVID-19 Vaccination

#### Model Testing

Confirmative factor analysis showed that perceptions fit the data well (CFI=0.98, TLI=0.90, and RMSEA=0.08). All the factor loadings were significant at *P<.*001, with β ranging from .23 to .73. The interpersonal-level variables also fit the data well (CFI=0.98, TLI=0.97, and RMSEA=0.02). All the factor loadings were significant at *P*<.001, with standardized coefficients ranging from .27 to .50. The hypothesized mediation model showed good fit to the data (CFI=0.96, TLI=0.94, and RMSEA=0.03).

#### Path Coefficients

**Figure 5:**
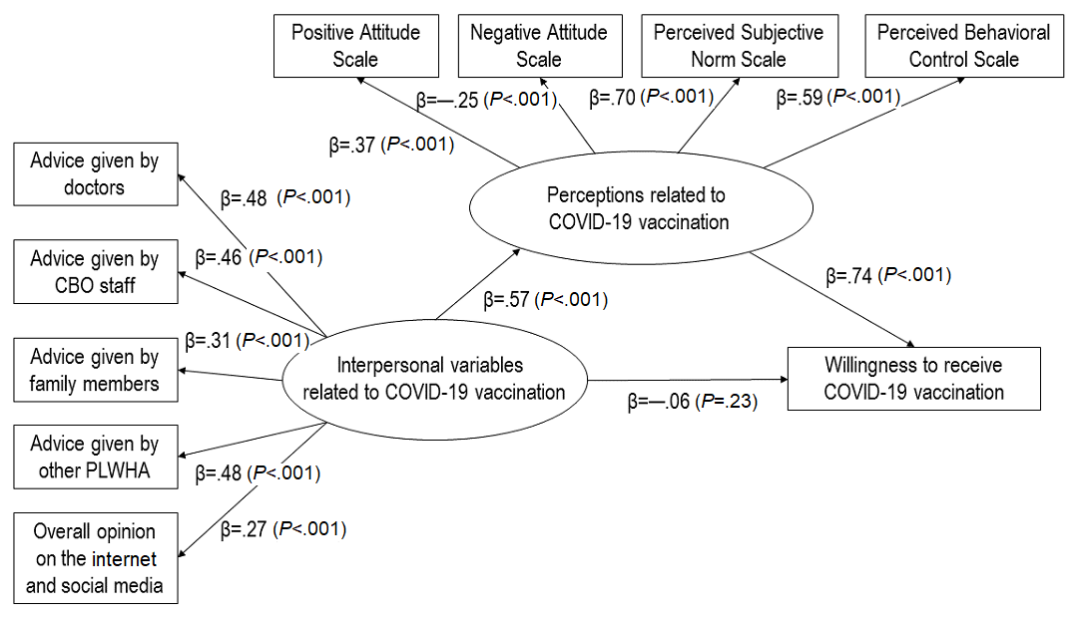
Mediation model with path coefficients. CBO: community-based organization; PLWHA: people living with HIV and AIDS.

Path analysis showed that interpersonal-level variables were positively associated with perceptions (B=4.72, β=.57, *P*<.001), while their association with willingness to receive COVID-19 vaccination was nonsignificant (B=–0.28, β=–.06, *P*=.23). Perceptions were positively associated with willingness to receive COVID-19 vaccination (B=0.43, β=.74, *P*<.001; Figure 5).

#### Mediation Effects

Bootstrap analysis showed that interpersonal-level variables (B=2.01, 95% CI 1.67-2.53; β=.43, 95% CI .37-.51; *P*<.001) were indirectly associated with willingness to receive COVID-19 vaccination via perceptions. Perceptions fully mediated the association between interpersonal-level variables and willingness to receive COVID-19 vaccination.

## Discussion

### Principal Findings

We found that willingness to receive COVID-19 vaccination among PLWHA is essential in the scale-up of COVID-19 vaccination among this group. The finding represents the latest estimate of willingness to receive COVID-19 vaccination among PLWHA in China and can be used to project future vaccine uptake in this group. We extended the existing literature by conducting this study in multiple cities in different geographic regions of China, with a large sample size, and examined the multiple-level factors that correlated with willingness to receive the vaccine.

We found that the level of willingness among our participants was relatively low (<60%). This level was lower than that of PLWHA in France and the United States as well as the general population in most parts of the world [19] and China (70%-90%) [26,27]. Since there is a gap between willingness and actual uptake, COVID-19 vaccination coverage among PLWHA would be even lower without effective interventions [32]. The above findings revealed COVID-19 vaccination hesitancy and highlighted a strong need to promote COVID-19 vaccination among PLWHA.

This study examined associated factors at all three levels suggested by the socioecological model; the findings could inform tailored interventions promoting COVID-19 vaccination among PLWHA. This was the first study to demonstrate that the individual-level variables (ie, perceptions related to COVID-19 vaccination) and interpersonal-level variables (ie, advice from others and information exposure on the internet and social media) were determinants of willingness among PLWHA. The findings extended the application of the socioecological model. More importantly, this study examined the potential mechanism of the associations between interpersonal interactions and willingness to receive COVID-19 vaccination. The results suggest that exposure to advice and information supporting PLWHA in receiving COVID-19 vaccination might enhance perceptions favoring COVID-19 vaccination, which, in turn, may increase their willingness to receive such vaccination. The significant mediation effect supported the mechanism proposed by the social learning theory [33].

This study also had numerous practical implications for developing tailored vaccination strategies for PLWHA. First, more attention should be given to PLWHA who are transgender persons, with chronic conditions, and who have severe AIDS-related symptoms, as these subgroups reported lower willingness. Transgender people are often marginalized, encountering difficulties to access health care services [34]. Future programs targeting PLWHA should consider including transgender-friendly vaccination services. Having AIDS-related symptoms was also associated with lower willingness. Since official opinions in China stated that the effectiveness of COVID-19 vaccination was lower for people with immunodeficiency [18], PLWHA might think that they may not benefit from COVID-19 vaccination. Health communication messages should clearly state that it is recommended that PLWHA receive COVID-19 vaccination if their chronic conditions are stable, regardless of AIDS-related symptoms.

Second, modifying perceptions related to COVID-19 vaccination is potentially useful in health promotion, as they were significantly associated with willingness in the expected directions. It is useful to increase positive attitudes toward COVID-19 vaccination, as this was a facilitator. Health communication messages should emphasize the physical and psychological benefits of COVID-19 vaccination. About half of the participants had concerns related to side effects, exposing PLWHA’s identities, and potential interactions between COVID-19 vaccines and HIV and ART. Having more concerns was associated with lower willingness. Testimonials on positive experiences shared by vaccinated PLWHA might be useful in reducing their concerns related to side effects and privacy. Health communication messages should also emphasize that there is no evidence showing that ART and COVID-19 vaccination would have negative impacts on each other [35]. Less than 40% of the participants perceived that medical professionals, CBO staff, family members, and friends would support them in taking up COVID-19 vaccination. Such perception was also a facilitator. Future programs should consider involving the significant others of PLWHA in order to create a subjective norm favoring COVID-19 vaccination uptake. It is also useful to enhance perceived behavioral control, as this was another facilitator. There is much room for improvement. Facilitating PLWHA in forming a plan to receive COVID-19 vaccination may be helpful for improving perceived behavioral control.

Third, the significant mediation effect of perceptions on the association between interpersonal-level variables and willingness to receive COVID-19 vaccination suggested that future programs should involve clinical doctors, CBO staff, friends and/or family members, and peers of PLWHA to give supportive advice. Health authorities should also disseminate clear recommendations for PLWHA to receive COVID-19 vaccination through official online channels, which are considered influential and credible sources by Chinese people [36]. These strategies may be useful to modify PLWHA’s perceptions and, in turn, increase their willingness to receive COVID-19 vaccination.

This study also had some limitations. First, policies and guidelines related to COVID-19 vaccination are changing rapidly. Our findings are most applicable to the early phase of COVID-19 vaccination implementation in China. Second, participants were recruited in large Chinese cities. Generalizations to PLWHA living in smaller cities or counties in China should be made cautiously. Third, we were not able to collect information from PLWHA who refused to participate in the study. PLWHA who refused to complete the survey may have different characteristics from the participants. Selection bias existed. Fourth, most items and scales used in this study were self-constructed based on those used in the general population. The internal validity of these scales was acceptable. However, external validation data were seldom available. Fifth, it was a limitation that we did not ask whether participants anticipated or had experienced challenges in making an appointment to receive the COVID-19 vaccine. The procedures to make an appointment are easy. Most Chinese people did not encounter difficulties when they were using the appointment system. We believe that the impact of anticipated and experienced challenges in making an appointment on PLWHA’s willingness to receive COVID-19 vaccination was limited. Sixth, we did not study PLWHA’s preferences for different types of COVID-19 vaccines. Although inactivated vaccines were the only available COVID-19 vaccines in China during the study period, it is worthwhile to look at their preferences for other types of vaccines (eg, mRNA or adenovirus vector vaccines) [37]. Moreover, the selection of the time frame for the history of other vaccinations (ie, past 3 years) was arbitrary. Furthermore, causality could not be established, as this was a cross-sectional study.

### Conclusions

In sum, PLWHA in China reported a relatively low willingness to receive COVID-19 vaccination compared to PLWHA in other countries and the general population in most parts of the world. Perceptions related to COVID-19 vaccination and interpersonal-level variables, such as receiving advice from others or information exposure through the internet and social media, were determinants of willingness. Information exposure on the internet and social media and interpersonal communications with doctors, CBO staff, friends, family members, and other PLWHA may be major sources of influence on PLWHA’s perceptions and willingness to receive COVID-19 vaccination. The study findings could be used to design tailored interventions with the aims of improving vaccination coverage and reduce risks of COVID-19 among PLWHA.

## Appendix

Multimedia Appendix 1 Frequency distribution of items measuring individual-level and interpersonal-level variables.

## Abbreviations

aOR: adjusted odds ratio
ART: antiretroviral therapy
CBO: community-based organization
CFI: comparative fit index
mRNA: messenger RNA
PLWHA: people living with HIV and AIDS
RMSEA: root mean square error of approximation
TLI: Tucker-Lewis index
WHO: World Health Organization

## References

[1] (2021). Coronavirus disease (COVID-19): COVID-19 vaccines and people living with HIV. World Health Organization.

[2] Bartsch SM, O'Shea KJ, Ferguson MC, Bottazzi ME, Wedlock PT, Strych U, McKinnell JA, Siegmund SS, Cox SN, Hotez PJ, Lee BY (2020). Vaccine efficacy needed for a COVID-19 coronavirus vaccine to prevent or stop an epidemic as the sole intervention. Am J Prev Med.

[3] Polack FP, Thomas SJ, Kitchin N, Absalon J, Gurtman A, Lockhart S, Perez JL, Pérez MG, Moreira ED, Zerbini C, Bailey R, Swanson KA, Roychoudhury S, Koury K, Li P, Kalina WV, Cooper D, Frenck RW, Hammitt LL, Türeci Ö, Nell H, Schaefer A, Ünal S, Tresnan DB, Mather S, Dormitzer PR, Şahin U, Jansen KU, Gruber WC, C4591001 Clinical Trial Group (2020). Safety and efficacy of the BNT162b2 mRNA Covid-19 vaccine. N Engl J Med.

[4] Voysey M, Clemens S, Madhi S, Weckx L, Folegatti P, Aley P, Angus B, Baillie VL, Barnabas SL, Bhorat QE, Bibi S, Briner C, Cicconi P, Collins AM, Colin-Jones R, Cutland CL, Darton TC, Dheda K, Duncan CJA, Emary KRW, Ewer KJ, Fairlie L, Faust SN, Feng S, Ferreira DM, Finn A, Goodman AL, Green CM, Green CA, Heath PT, Hill C, Hill H, Hirsch I, Hodgson SHC, Izu A, Jackson S, Jenkin D, Joe CCD, Kerridge S, Koen A, Kwatra G, Lazarus R, Lawrie AM, Lelliott A, Libri V, Lillie PJ, Mallory R, Mendes AVA, Milan EP, Minassian AM, McGregor A, Morrison H, Mujadidi YF, Nana A, O'Reilly PJ, Padayachee SD, Pittella A, Plested E, Pollock KM, Ramasamy MN, Rhead S, Schwarzbold AV, Singh N, Smith A, Song R, Snape MD, Sprinz E, Sutherland RK, Tarrant R, Thomson EC, Török ME, Toshner M, Turner DPJ, Vekemans J, Villafana TL, Watson MEE, Williams CJ, Douglas AD, Hill AVS, Lambe T, Gilbert SC, Pollard AJ, Oxford COVID Vaccine Trial Group (2021). Safety and efficacy of the ChAdOx1 nCoV-19 vaccine (AZD1222) against SARS-CoV-2: An interim analysis of four randomised controlled trials in Brazil, South Africa, and the UK. Lancet.

[5] Baden LR, El Sahly HM, Essink B, Kotloff K, Frey S, Novak R, Diemert D, Spector SA, Rouphael N, Creech CB, McGettigan J, Khetan S, Segall N, Solis J, Brosz A, Fierro C, Schwartz H, Neuzil K, Corey L, Gilbert P, Janes H, Follmann D, Marovich M, Mascola J, Polakowski L, Ledgerwood J, Graham BS, Bennett H, Pajon R, Knightly C, Leav B, Deng W, Zhou H, Han S, Ivarsson M, Miller J, Zaks T, COVE Study Group (2021). Efficacy and safety of the mRNA-1273 SARS-CoV-2 vaccine. N Engl J Med.

[6] Sadoff J, Gray G, Vandebosch A, Cárdenas V, Shukarev G, Grinsztejn B, Goepfert PA, Truyers C, Fennema H, Spiessens B, Offergeld K, Scheper G, Taylor KL, Robb ML, Treanor J, Barouch DH, Stoddard J, Ryser MF, Marovich MA, Neuzil KM, Corey L, Cauwenberghs N, Tanner T, Hardt K, Ruiz-Guiñazú J, Le Gars M, Schuitemaker H, Van Hoof J, Struyf F, Douoguih M, ENSEMBLE Study Group (2021). Safety and efficacy of single-dose Ad26.COV2.S vaccine against Covid-19. N Engl J Med.

[7] Shinde V, Bhikha S, Hoosain Z, Archary M, Bhorat Q, Fairlie L, Lalloo U, Masilela MS, Moodley D, Hanley S, Fouche L, Louw C, Tameris M, Singh N, Goga A, Dheda K, Grobbelaar C, Kruger G, Carrim-Ganey N, Baillie V, de Oliveira T, Lombard Koen A, Lombaard JJ, Mngqibisa R, Bhorat AE, Benadé G, Lalloo N, Pitsi A, Vollgraaff P, Luabeya A, Esmail A, Petrick FG, Oommen-Jose A, Foulkes S, Ahmed K, Thombrayil A, Fries L, Cloney-Clark S, Zhu M, Bennett C, Albert G, Faust E, Plested JS, Robertson A, Neal S, Cho I, Glenn GM, Dubovsky F, Madhi SA, 2019nCoV-501 Study Group (2021). Efficacy of NVX-CoV2373 Covid-19 vaccine against the B.1.351 variant. N Engl J Med.

[8] Hosein SR (2021). Encouraging results from the Pfizer-BioNTech COVID-19 vaccine in HIV-positive people. CATIE.

[9] Ruddy J, Boyarsky B, Bailey J, Karaba A, Garonzik-Wang J, Segev D, Durand CM, Werbel WA (2021). Safety and antibody response to two-dose SARS-CoV-2 messenger RNA vaccination in persons with HIV. AIDS.

[10] Woldemeskel B, Karaba A, Garliss C, Beck E, Wang K, Laeyendecker O, Cox AL, Blankson JN (2021). The BNT162b2 mRNA vaccine elicits robust humoral and cellular immune responses in people living with HIV. Clin Infect Dis.

[11] Frater J, Ewer KJ, Ogbe A, Pace M, Adele S, Adland E, Alagaratnam J, Aley PK, Ali M, Ansari MA, Bara A, Bittaye M, Broadhead S, Brown A, Brown H, Cappuccini F, Cooney E, Dejnirattisai W, Dold C, Fairhead C, Fok H, Folegatti PM, Fowler J, Gibbs C, Goodman AL, Jenkin D, Jones M, Makinson R, Marchevsky NG, Mujadidi YF, Nguyen H, Parolini L, Petersen C, Plested E, Pollock KM, Ramasamy MN, Rhead S, Robinson H, Robinson N, Rongkard P, Ryan F, Serrano S, Tipoe T, Voysey M, Waters A, Zacharopoulou P, Barnes E, Dunachie S, Goulder P, Klenerman P, Screaton GR, Winston A, Hill AVS, Gilbert SC, Pollard AJ, Fidler S, Fox J, Lambe T, Oxford COVID Vaccine Trial Group (2021). Safety and immunogenicity of the ChAdOx1 nCoV-19 (AZD1222) vaccine against SARS-CoV-2 in HIV infection: A single-arm substudy of a phase 2/3 clinical trial. Lancet HIV.

[12] Madhi S, Koen A, Fairlie L, Cutland C, Ballie V, Padayachee S ChAdOx1 nCoV-19 (AZD1222) vaccine in people living with and without HIV. Research Square..

[13] United States Department of Health and Human Services (2021). Guidance for COVID-19 and people with HIV. Guidelines for the Prevention and Treatment of Opportunistic Infections in Adults and Adolescents with HIV.

[14] (2021). British HIV Association.

[15] ASHM COVID-19 Taskforce​ (2021). Statement from the ASHM COVID-19 Taskforce regarding the prioritization of COVID-19 vaccines for people living with HIV. Australasian Society for HIV, Viral Hepatitis and Sexual Health Medicine.

[16] (2021). COVID-19 vaccines for moderately to severely immunocompromised people. Centers for Disease Control and Prevention.

[17] Chapter of Infectious Disease Physicians (2021). Consensus Statement on the Expansion of Eligibility for COVID-19 Vaccination for People Living With HIV in Singapore.

[18] National Health Commission of the People's Republic of China Technical Guideline on COVID-19 Vaccination (1st Edition).

[19] Sallam M (2021). COVID-19 vaccine hesitancy worldwide: A concise systematic review of vaccine acceptance rates. Vaccines (Basel).

[20] COCONEL Group (2020). A future vaccination campaign against COVID-19 at risk of vaccine hesitancy and politicisation. Lancet Infect Dis.

[21] Wang Z, She R, Chen X, Li L, Li L, Huang Z, Lau JTF (2021). Parental acceptability of COVID-19 vaccination for children under the age of 18 years among Chinese doctors and nurses: A cross-sectional online survey. Hum Vaccin Immunother.

[22] Vallée A, Fourn E, Majerholc C, Touche P, Zucman D (2021). COVID-19 vaccine hesitancy among French people living with HIV. Vaccines (Basel).

[23] Bogart L, Ojikutu B, Tyagi K, Klein D, Mutchler M, Dong L, Lawrence SJ, Thomas DR, Kellman S (2021). COVID-19 related medical mistrust, health impacts, and potential vaccine hesitancy among Black Americans living with HIV. J Acquir Immune Defic Syndr.

[24] McLeroy KR, Bibeau D, Steckler A, Glanz K (1988). An ecological perspective on health promotion programs. Health Educ Q.

[25] Pan Y, Fang Y, Xin M, Dong W, Zhou L, Hou Q, Li F, Sun G, Zheng Z, Yuan J, Wang Z, He Y (2020). Self-reported compliance with personal preventive measures among Chinese factory workers at the beginning of work resumption following the COVID-19 outbreak: Cross-sectional survey study. J Med Internet Res.

[26] Wang J, Jing R, Lai X, Zhang H, Lyu Y, Knoll MD, Fang H (2020). Acceptance of COVID-19 vaccination during the COVID-19 pandemic in China. Vaccines (Basel).

[27] Zhang KC, Fang Y, Cao H, Chen H, Hu T, Chen Y, Zhou X, Wang Z (2021). Behavioral intention to receive a COVID-19 vaccination among Chinese factory workers: Cross-sectional online survey. J Med Internet Res.

[28] Wong MC, Wong EL, Huang J, Cheung AW, Law K, Chong MK, Ng RW, Lai CK, Boon SS, Lau JT, Chen Z, Chan PK (2021). Acceptance of the COVID-19 vaccine based on the health belief model: A population-based survey in Hong Kong. Vaccine.

[29] Wang K, Wong EL, Ho K, Cheung AW, Yau PS, Dong D, Wong SY, Yeoh E (2021). Change of willingness to accept COVID-19 vaccine and reasons of vaccine hesitancy of working people at different waves of local epidemic in Hong Kong, China: Repeated cross-sectional surveys. Vaccines (Basel).

[30] Yu Y, Lau JT, Lau MM, Wong MC, Chan PK (2021). Understanding the prevalence and associated factors of behavioral intention of COVID-19 vaccination under specific scenarios combining effectiveness, safety, and cost in the Hong Kong Chinese general population. Int J Health Policy Manag.

[31] Lau JT, Wang Z, Kim Y, Li J, Gu J, Mo PK, Wang X (2017). Low sustainability, poor governance, and other challenges encountered by grassroots non-governmental organizations targeting HIV prevention for men who have sex with men in China - A nation-wide study. AIDS Care.

[32] Michie S, Johnston M, Francis J, Hardeman W, Eccle M (2008). From theory to intervention: Mapping theoretically derived behaviorual determinants to behaviour change techniques. Appl Psychol.

[33] Moreno MA, Whitehill JM (2014). Influence of social media on alcohol use in adolescents and young adults. Alcohol Res.

[34] Poteat T, Wirtz AL, Radix A, Borquez A, Silva-Santisteban A, Deutsch MB, Khan SI, Winter S, Operario D (2015). HIV risk and preventive interventions in transgender women sex workers. Lancet.

[35] (2021). COVID-19 Vaccines and HIV.

[36] Pan Y, Xin M, Zhang C, Dong W, Fang Y, Wu W, Li M, Pang J, Zheng Z, Wang Z, Yuan J, He Y (2020). Associations of mental health and personal preventive measure compliance with exposure to COVID-19 information during work resumption following the COVID-19 outbreak in China: Cross-sectional survey study. J Med Internet Res.

[37] Zewude B, Habtegiorgis T (2021). Willingness to take COVID-19 vaccine among people most at risk of exposure in Southern Ethiopia. Pragmat Obs Res.

