# Supplementary material for: Willingness to Receive COVID-19 Vaccination Among People Living With HIV and AIDS in China: Nationwide Cross-sectional Online Survey
Source: JMIR Public Health Surveill. 2021 Oct 21;7(10):e31125. doi: 10.2196/31125 (PMC8534487; doi:10.2196/31125)
Supplement: Multimedia Appendix 1 [file publichealth_v7i10e31125_app1.docx]

Multimedia appendix 1. Frequency distribution of items measuring individual-level and interpersonal-level variables

|  | N | % |
| --- | --- | --- |
| Willingness to receive free COVID-19 vaccination |  |  |
| No (very unlikely/unlikely/neutral) | 1100 | 42.8 |
| Yes (likely/very likely) | 1470 | 57.2 |
|  |  |  |
| **Socio-structural-level variables** |  |  |
| Individuals could make an appointment to receive COVID-19 vaccination during the study period |  |  |
| No | 1578 | 61.4 |
| Yes | 992 | 38.6 |
| There was a shortage of COVID-19 vaccine in the city where the participants were living during the study period |  |  |
| No | 1729 | 67.3 |
| Yes | 841 | 32.7 |
| Whether participants belonged to any priority groups to receive COVID-19 vaccination in their cities during the study period |  |  |
| No | 2082 | 81.0 |
| Yes | 488 | 19.0 |
|  |  |  |
| **Individual-level variables** |  |  |
| ***Perceptions and attitudes toward COVID-19 vaccination*** |  |  |
| Positive attitudes toward COVID-19 vaccination (% agree or strongly agree) |  |  |
| COVID-19 vaccination is effective in improving immune function | 1081 | 42.1 |
| COVID-19 vaccination is effective in reducing your risk of SARS-CoV-2 infection | 1718 | 66.8 |
| COVID-19 vaccination is effective in reducing mortality caused by COVID-19 | 1494 | 58.1 |
| COVID-19 vaccination is effective in reducing severity of COVID-19 | 1540 | 59.9 |
| Taking up COVID-19 vaccination can make you feel relieved | 1595 | 62.1 |
| *Positive Attitude Scale ^a^, mean (SD)* | *18.4* | *(4.8)* |
|  |  |  |
| Negative attitudes toward COVID-19 vaccination (% agree or strongly agree) |  |  |
| COVID-19 vaccination has severe side effects | 1124 | 43.7 |
| COVID-19 vaccination uptake has significant negative influence on effectiveness of ART | 1536 | 59.8 |
| You have concerns about the risk of exposing your PLWHA identity when taking up COVID-19 vaccination | 1776 | 69.1 |
| HIV infection has significant negative influence on effectiveness of COVID-19 vaccination | 1488 | 57.9 |
| The side effects of COVID-19 vaccination are more severer for PLWH than those without HIV infection | 1549 | 60.3 |
| *Negative Attitude Scale ^b^, mean (SD)* | *18.6* | *(5.2)* |
|  |  |  |
| Subjective norms related to COVID-19 vaccination (% agree or strongly agree) |  |  |
| Your family members will support you to take up COVID-19 vaccination | 1015 | 39.5 |
| Your HIV-infected friends will support you to take up COVID-19 vaccination | 648 | 25.2 |
| Medical professionals will support you to take up COVID-19 vaccination | 892 | 34.7 |
| CBO workers will support you to take up COVID-19 vaccination | 866 | 33.7 |
| *Perceived Subjective Norm Scale ^c^, mean (SD)* | *13.3* | *(2.4)* |
|  |  |  |
| Perceived behavioral control to take up COVID-19 vaccination (% agree or strongly agree) |  |  |
| You will take up COVID-19 vaccination even if it will interrupt your daily routine | 828 | 32.2 |
| You will take up COVID-19 vaccination even when you do not feel well | 570 | 22.2 |
| You will take up COVID-19 vaccination even if the side-effects would affect your daily activities | 614 | 23.9 |
| You will take up COVID-19 vaccination even if HIV infection would reduce its effectiveness | 775 | 30.2 |
| You will take up COVID-19 vaccination even if it will reduce effectiveness of ART | 524 | 20.4 |
| *Perceived Behavioral Control Scale ^d^, mean (SD)* | *12.9* | *(6.1)* |
|  |  |  |
| **Interpersonal-level variables** |  |  |
| Advice from doctors regarding COVID-19 vaccination |  |  |
| Against taking up COVID-19 vaccination | 140 | 5.4 |
| No advice/neutral | 2017 | 78.5 |
| Supportive to take up COVID-19 vaccination | 413 | 16.1 |
| *Mean (SD)* | *2.1* | *(0.5)* |
| Advices from CBO staff regarding COVID-19 vaccination |  |  |
| Against taking up COVID-19 vaccination | 88 | 3.4 |
| No advice/neutral | 2194 | 85.4 |
| Supportive to take up COVID-19 vaccination | 288 | 11.2 |
| *Mean (SD)* | *2.1* | *(0.4)* |
| Advices from friends and family members regarding COVID-19 vaccination |  |  |
| Against taking up COVID-19 vaccination | 36 | 1.4 |
| No advice/neutral | 2485 | 96.7 |
| Supportive to take up COVID-19 vaccination | 49 | 1.9 |
| *Mean (SD)* | *2.0* | *(0.2)* |
| Advices from other PLWHA regarding COVID-19 vaccination |  |  |
| Against taking up COVID-19 vaccination | 131 | 5.1 |
| No advice/neutral | 2276 | 88.6 |
| Supportive to take up COVID-19 vaccination | 163 | 6.3 |
| *Mean (SD)* | *2.0* | *(0.3)* |
| Overall opinion regarding COVID-19 vaccination for PLWHA on Internet/social media |  |  |
| Against taking up COVID-19 vaccination | 173 | 6.7 |
| No advice/neutral | 2131 | 82.9 |
| Supportive to take up COVID-19 vaccination | 266 | 10.4 |
| *Mean (SD)* | *2·0* | *(0.4)* |

SD, standard deviation·

^a^ Positive Attitude Scale, 5 items, maximum value = 25; Cronbach’s alpha: 0.83, one factor was identified by exploratory factor analysis, explaining for 61.1% of total variance

^b^ Negative Attitude Scale, 5 items, maximum value = 25; Cronbach’s alpha: 0.87, one factor was identified by exploratory factor analysis, explaining for 66.3% of total variance

^c^ Perceived Subjective Norm Scale, 4 items, maximum value = 20; Cronbach’s alpha: 0.84, one factor was identified by exploratory factor analysis, explaining for 63.4% of total variance

^d^ Perceived Behavioral Control Scale, 5 items, maximum value = 25; Cronbach’s alpha: 0.92, one factor was identified by exploratory factor analysis, explaining for 76.4% of total variance
